# Personal News Items

**Published:** 1914-12

**Authors:** 


### Personal News Items.

Dr. and Mrs. E. M. Stipp, of Burnet, announce the arrival of a baby girl on the 10th.

Dr. Joe Becton, of Greenville, attended the Southwest Texas Medical meeting in Galveston.

Dr. G. W. Yeakley, of Bowie, has returned from Chicago, where he has been taking a post-graduate course.

Dr. Frank Seay, of Georgetown, underwent an operation for appendicitis at the Temple Sanitarium recently.

Dr. E. O. Deal, of Mertzon, attended the meeting of the Tom Green County Medical Society at San Angelo.

Drs. Durham, Wysong, Hall and Curry of Hico attended the session of the Hamilton County Medical Society, at Hamilton.

Dr. and Mrs. E. M. Wood, of Georgetown, attended the unveiling ceremonies of the W. O. W. monument at Florence.

Dr. J. E. Gilcreest, of Gainesville, has honored the National Association of Surgeons by becoming a member of that body.

Dr. W. T. Thackery, of Fowlerton, has recently had X-ray treatment for a cancer on the tongue. The doctor was much benefited.

Dr. John F. Bailey, of Waco, has returned to his home from Houston, having recovered from an illness of nearly two months.

The will of George Hammond left about three million dollars for the establishment of an immense charity hospital in Houston, Texas.

Dr. A. S. Epperson and Dr. G. B. Taylor, of Cameron, attended the meeting of the Tri-State Medical Association in Galveston.

Dr. C. M. Rosser, of Dallas, delivered an address before the Baptist General Convention of Texas, which met recently in Abilene. Dr. Rosser is professor of surgery of the Baylor University.

Dr. J. B. Cranfill, of Dallas, has been elected President of the Wear Cotton Goods Club. This is an organization that should spread all over the country, and especially should it be strong in the South.

The State Board of Medical Examiners recently met in Waco. About forty applicants for State licenses were examined by the board. Several foreign countries and a number of States were represented.

Dr. H. L. Hilgartner, of Austin, attended the meeting of the Southern Association of Surgeons. From Richmond Dr. Hilgartner goes to Baltimore and other Eastern clinics to visit, returning home about December 1st.

Dr. R. S. Aigueir, formerly with the St. Vincent's Hospital staff at Sherman, has joined the staff of Parkland Hospital in Dallas. Dr. R. J. Johnson has resigned from the city staff and has taken a practice in Louisiana.

Dr. D. J. Jenkins, of Daingerfield, has been selected by the management of the Northeast Texas Fair at Pittsburg to act as one of the judges in the Better Babies Contest, which will be one of the leading features of the fair.

Dr. R. J. Brackenridge and Mrs. Brackenridge have returned from New York, where they have been on a visit during the last two months. Dr. Brackenridge will spend a few days in San Antonio and will then return to Austin for the winter.

Dr. Burns, of Cuero, left November 10th for the East and North to attend the Southern Medical Convention; from there he will go to some of the big New York hospitals. He will conclude his trip with a stay at Mayo Brothers' Sanitarium, Rochester, Minn., where he will do special research work.

Dr. Walter F. McCaleb, of San Antonio, who is soon to assume his duties as director of the new regional reserve bank at Dallas, was honored by the members of the Travis Club and presented with a gold watch and a life membership as a token of their esteem. He was vice-president of the organization.

Several Dallas physicians were honored by being named fellows of the American College of Surgery. The Dallas physicians so recognized include the following: Drs. R. S. Yancey, J. B. Smoot, E. M. Lott, H. M. Doolittle and E. Dunlap. All of them went to Washington, where their new honors were conferred.

Dr. White, Superintendent of the Insane Asylum of San Antonio, recently delivered an interesting and instructive lecture on "Nervous Diseases" to the Mothers' Club of Floresville. Those who heard the lecture were well paid for attending, and reported that much valuable information was gathered from the important points brought out so clearly by Dr. White.

Drs. R. W. Knox, Houston; E. H. Cary, M. M. Carrick, Elbert Dunlap, Dallas; Frank P. Boyd, C. H. Harris, Fort Worth; Connolley and Burgess, Waco, attended the session of the Southern Medical Association, and helped to capture the 1915 meeting for Dallas. They announce that Dr. W. R. Rodman, President of the American Medical Association, has accepted an invitation to address the State Medical Association next year. Dr. Rodman is a Texan, having begun his medical career at Abilene.

That a cure for epilepsy through a surgical operation on the intestines has been demonstrated in many cases was announced at the meeting of the Mississippi Valley Medical Association at Cincinnati by Dr. Charles A. L. Reed, the discoverer. Dr. Reed held that the majority of cases of epilepsy are caused by poisons assembled by the human system from the intestines.

Dr. Conrad Frey, of San Antonio, is now located in Comfort, where he went to relieve Dr. C. C. Jones, who has gone to Chicago, where he will spend a month or two taking some special courses. Dr. Frey is a German, and speaks that language fluently.

According to the United States Geological Survey, ichthyol has been found in Burnet, Texas, the only place in the United States, the deposits covering a tract of about 500 acres. Control of the property has been secured by New York interests, and the deposits will be developed under the direction of Dr. Polatsik. The price of ichthyol is said to have advanced considerably since the European war started, so the development of the Texas product is likely to prove profitable.

Dallas book dealers have received supplies of a new book by D. T. Atkinson, M. D., a well known Dallas eye, ear, nose and throat specialist. The title of the volume is "Adenoids and Kindred Perils of School Life," and is declared to be one of the most comprehensive volumes ever written on the subject. The book is especially written for the use and information of parents and teachers. The wordage is entire free of technical terms so as to be easily read and understood by the layman. The volume is dedicated to "The American School Children." Dr. D. T. Atkinson, the author, is editor of the eye, ear nose and throat department of the *Texas Medical News,* and is a member of the staff of St. Paul's Sanitarium of Dallas.

The microbe which causes gangrene in bullet and shrapnel wounds has been discovered by Drs. James Scarlett and Georges des Jardins of the American Ambulance Service. Previously initial cultures all were impure, leading to the belief of scientists that the disease was caused not by a single germ, but by a combination of germs. After much research and experimentation on horses and guinea pigs, a single bacillus has been discovered and isolated and the serum is now being prepared by the Pasteur Institute. The discovery is expected in medical circles to have world-wide importance. The serum is being injected into patients on the battlefield in the early stages of infection, obviating amputations and preventing a great loss of life.

Dr. Wilbert of the United States Public Health Service says: "The people of the United States annually spend more than $500,000,000 for medicine, and by far the greater bulk of the medicine purchased is consumed haphazardly and not under the direct supervision of experts, whose knowledge would tend to prevent harmful intoxication and untoward results from the injection of potent and in many instances dangerously harmful preparations. One instance cited by the doctor is striking. He finds that enough quinine was imported into the United States in 1913 to give every man, woman and child from twenty-five to thirty doses each year—a total of 2,065,000,000 average doses. This family standby he declares to be the cause of gastric catarrh, even when given in small doses, so what happens when the public gets these two billion, sixty-five million doses may be inferred. The report contains much specific and shocking detail as to the use of habit-forming drugs and alcohol in patent medicines.

